# Infant feeding practices and associated factors among HIV-positive mothers of infants aged 0–6 months at public health facilities in Addis Ababa, Ethiopia

**DOI:** 10.1186/s41043-024-00496-5

**Published:** 2024-02-20

**Authors:** Zewdu Minwuyelet Gebremariam, Genanew Getahun, Addisu Sahile, Yared Kejela, Yeworkwuha Getachew, Fasil Sisay

**Affiliations:** 1 Menelik II Medical and Health Science College, City Administration of Addis Ababa Health Bureau, Addis Ababa, Ethiopia; 2 City Administration of Addis Ababa Health Bureau, Addis Ababa, Ethiopia; 3 Unity University, Addis Ababa, Ethiopia

**Keywords:** Exclusive breast-feeding, HIV-positive mothers, Infant feeding practice, Exclusive replacement feeding, Ethiopia

## Abstract

**Background:**

In the era of HIV infection, exclusive breast-feeding highly recommended for infants aged less than 6 months. Avoidance of exclusive breast-feeding by HIV-infected mothers recommended when replacement feeding is acceptable, feasible, affordable, sustainable and safe. The prevalence of exclusive breast-feeding has remained very low worldwide. Despite this fact, there is limited information on infant feeding practices of HIV-positive mothers and factors that affect the practice in the current study area.

**Objective:**

This study assessed the magnitude of infant feeding practice and associated factors among HIV-positive mothers of infants aged 0–6 months at public health facilities in Addis Ababa, Ethiopia.

**Methods:**

A multicenter facility-based cross-sectional study design was employed among a total of 397 study participants. The study participants were selected using a simple random sampling technique. The completeness of the data was checked, coded, cleaned and entered into Epi-data version 4.6 software, and exported to SPSS version 24 for analysis. Descriptive statistics and Binary logistic regression model were employed for the analysis with adjusted odds ratio (AOR) with a 95% CI and a *P* value ≤ 0.05 to determine the strength of association between infant feeding practice and its independent factors.

**Results:**

The overall magnitude of appropriate infant feeding practice among HIV-positive mothers was 82.6% (95% CI 80.9–88.2). Good knowledge of mother’s toward infant feeding (AOR: 1.26, 95%, CI 1.11–3.34), better household monthly income, ≥ 6001 Ethiopian birr (AOR: 1.62, 95% CI 1.33–5.14) and favorable attitude of mother’s toward infant feeding (AOR: 1.71, 95% CI 1.01–2.92) were statistically significant associated factors with the recommended way of infant feeding practice.

**Conclusions and recommendations:**

Hence, the current study area is the capital city of the Ethiopia, where a relatively educated population lived in, there was an opportunity for better income, and appropriate infant feeding practice among HIV-positive mothers was found slightly higher than even the overall national target (70%) that was planned by 2020. Therefore, different stakeholders should develop strategic plan to excel females’ education coverage and thereby their knowledge and attitude toward infant feeding to fully eradicate mother-to-child transmission of diseases.

## Introduction

Appropriate infant feeding practice is essential for their health, proper growth and development [1, 2]. Therefore, mothers of children under the age of 6 months are recommended to practice appropriate infant feeding so as to achieve optimal nutrition outcomes in a population [1, 3–5]. Infant feeding practice under the age of 6 months can be categorized as recommended if it is to be either exclusive breast-feeding or replacement feeding and not recommended to use mixed feeding practice (MIF) [6]. This is because breast-feeding is considered the safest source of nutrition for the majority of infants [7, 8], and therefore, it has to be initiated as early as possible within an hour after delivery and continued exclusively for the first 6 months of life [1–5].

According to the World Health Organization report, mothers with a confirmed human immune virus (HIV) infection and whose infants are HIV-uninfected or of unknown HIV status should exclusively breast-feed their infants for the first 6 months of life and only stop when a nutritionally adequate and safe diet without breast milk can be provided [9, 10]. Moreover, HIV-positive mothers need to take antiretroviral therapy (ART) consistently throughout the breast-feeding period so that the risk of transmitting HIV to their children remains extremely low [9]. HIV-infected mothers would have to practice ERF only when replacement feeding is acceptable, feasible, acceptable, sustainable and safe (AFASS). However, the majority of mothers living in resource-limited countries cannot fulfill it [6]. In countries with high incomes, breast-feeding avoidance is recommended for preventing postnatal transmission of HIV; however, in low- and middle-income countries exclusive replacement feeding (formula use) can be dangerous because of issues of its acceptability, feasibility, affordability, sustainability or safe use of formula feeding [6, 10]. Hence, avoiding breast-feeding can possibly eliminate the risk of mother-to-child HIV transmission through exclusive replacement feeding, but it may cost the life of an infant because of the potential introduction of inappropriate feeding practices [11].

Due to the implementation of prevention of mother-to-child transmission (PMTCT) services across the globe, around 1.4 million HIV infections were prevented between 2010 and 2018 [12]. However, it was not 100% effective as it worked only for children whose mothers had access to antenatal care and PMTCT services [13]. According to the Ethiopian Public Health Institute (EPHI), the mothers who need PMTCT service in Ethiopia in 2019 was estimated to be 19,110. However, those who received PMTCT service were about 14,149. In addition, PMTCT coverage for the year was 74.04% with the final mother-to-child transmission (MTCT) rate of 16.90%. In addition, mothers in need of the service in Addis Ababa city were estimated to be 2352; while mothers who received the service were about 2307, the coverage was 98.08% with a final MTCT rate of 8.53% [14]. The target for elimination of HIV is reducing the final HIV transmission rate to 5% or less among breast-feeding mothers and to 2% or less among non-breast-feeding mothers by 2020 [12] and zero new infections by 2030 [13].

According to different studies conducted in different areas, there are several factors that affect the feeding practices of HIV-positive mothers of children younger than 6 mothers. These factors include socio-demographic characteristics, economic factors, maternal health and obstetric factors, awareness of appropriate infant feeding practices or exposure to information and attitude toward infant feeding. Socio-demographic characteristics include maternal age, maternal educational status, husband educational status, household income and employment status of mothers associated with the feeding practices of HIV-positive mothers [6, 15–18]. Maternal health and obstetric factors include antenatal care (ANC), the number of ANC visits, the mode and place of delivery, disclosure of HIV status to the spouse, the condition of maternal health and breast problems, which also influence practice of infant feeding [6]. Moreover, infant-related factors, including infant illness or mouth ulcer, pre-lacteal feeding status [6, 19], maternal knowledge and exposure to information [8, 20] and maternal attitude [21], influence overall infant feeding practice.

Infants who are not exclusively breast-fed are 15 times more likely to die from pneumonia and 11 times more likely to die from diarrhea than children who are exclusively breast-fed [10]. In addition, factors that influence the overall infant feeding practice among HIV-positive mothers vary from area to area particularly between developed and low- and middle-income countries, where there is a difference in adaptability, feasibility, acceptability, sustainability and safe use of exclusive replacement feeding practices. Therefore, the aim of this study was to assess the magnitude and factors that influence infant feeding practice in the current study area.

## Methods

### Study settings, period and design

The study was carried out in multicenter health facilities in Addis Ababa, the capital of Ethiopia, and the diplomatic center of Africa. So, it hosts a number of international organizations, such as the headquarters of African Union (AU) and the United Nations Economic Commission for Africa (UNECA). Due to its location and status, several people come to the city in search of employment opportunities and services. The city has three layers of administration, including city administration, eleven subcities called kifle-ketema and 116 woredas at the lowest administrative units (40). In the city, there are 12 Public heath hospitals (six federal hospitals and six regional hospitals) and 100 public health centers. Among these, 30% of these health facilities (*n* = 30) and 30% of public health hospitals (*n* = 4), namely Zewditu, Ghandi, St. Peter and ALERT Hospitals, were selected randomly.

A cross-sectional study design was employed among HIV-positive mothers who had less than 6 months of aged infants and attending PMTCT and/or on ART services at randomly selected 34 public health facilities in Addis Ababa city from August 1, 2022, to August 30, 2022.

### Population

The source population was all HIV-positive mothers who had less than 6 months of aged infants and attending PMTCT and/or on ART services at all health facilities in Addis Ababa, whereas randomly selected HIV-positive mothers who had less than 6 months of aged infants and attended PMTCT and/or on ART services during the study period were study population.

#### Sample size determination and sampling procedures

The sample size was calculated using single population proportion formula by considering the assumptions of confidence interval Z score standard value with a confidence level at 95%, proportion (*P*) of recommended infant feeding practice in Gulele subcity (37.4%) and *d*^2^ (marginal of error) 5% [22]. With this assumption, the calculated sample was 359, and by considering 10% non-response rate, the final sample size become 413. The study subjects were selected from each health facility based on simple random sampling techniques following probability proportional to size of study subjects served. Accordingly, a total of 358 and 55 study participants were selected from 30 health centers and four public health hospitals [Zewditu (*n* = 18), Ghandi (*n* = 14), St. Peter (*n* = 10) and ALERT (*n* = 13)]. The study subjects were all HIV-positive mothers who had infants aged between 0 and 6 months, and fulfill the eligibility criteria were randomly selected during the study period.

#### Eligibility criteria

The study included HIV-positive mothers who had infants aged between 0 and 6 months lived in Addis Ababa for more than 6 months prior to the study period, volunteer to participant in the study were included, where as those HIV-positive mothers with mental health problem and severe ills and unable to respond or communicate were excluded from the study.

### Study variables and definitions

Infant feeding practice was the dependent variable, and it was measured dichotomously as appropriate and non-appropriate infant feeding practices. The variable was measured as appropriate infant feeding practice if HIV-positive mothers and whose infants aged between 0 and 6 months feeding their infant either exclusive breast-feeding or exclusive replacement (formula) feeding, whereas it is inappropriate if the feed their infants in a mixed way before 6 months of infants age. Independent variables that contained socio-demographic and economic characteristics included the age, marital status, educational status of the mother, occupational status of both parents and their income; maternal health- and obstetrics-related factors such as antenatal care, number of ANC visits, mode of delivery, place of delivery, disclosure of HIV status to partner, postnatal care, maternal knowledge and maternal attitude toward infant feeding practices were measured; and infant-related factors such as timely initiation of infant breast-feeding and pre-lacteal feeding status were measured as independent factors. Most independent factors were measured as a dichotomous: presence (yes) or absence (no) of responses, whereas knowledge of mothers was measured as “good knowledge” when mothers who scored above the median value for knowledge questions, whereas mothers who scored below the median were considered to have “poor knowledge” [11]. Mothers’ attitude toward infant breast-feeding was measured as “appropriate attitude” and “inappropriate attitude” toward infant feeding practice. Accordingly, mothers who scored above the median for attitude questions were considered to have “favorable attitude,” whereas mothers who scored below the median were considered to have “unfavorable attitude” [6]. The data collection tool was organized after a rigorous review of related studies.

### Data quality management and statistical analysis

The quality of the data was assured throughout the research phases from inception of the research tool development to the report phase. Prior to data collection, the data collection tool was validated by inculcating experts’ opinion, and then, pretest was done in 5% of the total sample size in health facilities different from the current study area. In addition, 3-day training was given for data collectors and supervisors on the research objective, methods, data collection instrument, data collection technique, data collection procedure and the relevant ethical issues. During data collection, completeness of the interviewer administered structured questionnaires was checked by the supervisors and the principal investigators. After data collection, data were coded, entered using Epi Info 7 and then transferred into SPSS version 24 statistical packages for analyses. A binary logistic regression was done to determine the strength of association between the independent and independent variables. So, variables that had a *p* value less than 0.2 in the bi-variable logistic regression analysis were entered into a multivariable logistic regression to adjust the effect of confounders on the outcome variables. Multivariable logistic regression models were fitted to determine the presence of an association between the dependent and independent variables at a *p* value of 0.05 and an AOR with a 95% confidence interval.

### Ethical considerations

The researchers secured ethical approval from Menelik II Medical and Health Science College and Addis Ababa Health Bureau, Addis Ababa public health research and emergency management directorate. Permission letter was secured from the randomly selected public health facilities administrators. Informed written consent was obtained from each study participant before data collection.

## Results

### Socio-demographic and economic characteristics

A total of 413 HIV-positive mothers of infants aged 0–6 months attending PMTCT or ART services at public health facilities in Addis Ababa were included in this study with response rate of 96%. One hundred forty seven (37%) of HIV-infected mothers were within the age group from 25 to 29 years, and their mean age was 30.5 (SD ± 4.524) years. Three hundred thirty four (84.1%) of HIV-infected mothers were married, about 12.1% (*n* = 48) had no formal education, whereas 13 (3.3%) of spouses were unable to read and write (Table 1).

**Table 1:** Socio-demographic and economic characteristic of HIV-positive mothers’ of infants aged 0–6 months attended in public health facilities in Addis Ababa, Ethiopia, 2022 (*n* = 397)

Variable	Category	Frequency (*n*)	Percentage (%)
Mather’s age category	20–24	33	8.3
	25–29	147	37
	30–34	129	32.5
	35–39	75	18.9
	40–44	13	3.3
Marital status	Single	13	3.3
	Married	334	84.1
	Separated	42	10.6
	Divorced	5	1.3
	Widowed	3	0.8
Education status of the mother	No formal education	48	12.1
	Can read and write	72	18.1
	Primary	107	27
	Secondary	112	28.2
	College and above	58	14.6
Education status of the father	No formal education	13	3.3
	Can read and write	24	6.0
	Primary	89	22.4
	Secondary	104	26.2
	College and above	167	42.1
Occupation of mother	Governmental employee	86	21.7
	Private org. employee	119	30
	Housewife	192	48.3
Occupation of her spouse	Governmental employee	147	37
	NGO employee	29	7.3
	Private employee	117	29.5
	Daily laborer	59	14.9
	Merchant	36	9
	Others	9	2.3
Monthly income of the household (EBr)	< 3000	165	41.6
	3001–6000	115	28.9
	> 6001	117	29.5

### Maternal health- and obstetrics-related factors

About 22% (*n* = 86) of HIV-infected mothers responded that they had no plan for their last pregnancy; three hundred ninety (98.2%) study participants had at least one antenatal care during their last pregnancy. Regarding place and mode of delivery, most of them, 394 (99%) got health institutional delivery service and about 11% (*n* = 43) were delivered through caesarian section. The majority of the three hundred fifty one HIV-positive mothers (88%) had postnatal care services during their last pregnancy. About 74% (*n* = 292) and 24.9% (*n* = 99) of the respondents knew their HIV status before and during their last pregnancy, respectively. Ninety one percent of the respondents disclosed their HIV status to their spouses, 365 (91.9%) (Table 2).

**Table 2:** Maternal health- and obstetrics-related factors HIV-positive mothers of children 0–6 months attended in Public health facilities in Addis Ababa, Ethiopia, 2022 (*n* = 397)

S. no	Variable	Category	Frequency	Percentage
1	Was your last pregnancy planned?	Yes	311	78.3
		No	86	21.7
2	ANC follow-up for your last pregnancy	Yes	390	98.2
		No	7	1.8
3	Time of first ANC visit (*N* = 390)	First trim	165	42.3
		Second trim	185	47.4
		Third trim	40	10.3
4	Number of ANC visits during the last pregnancy (N = 390)	Once	55	14.1
		Twice	26	6.6
		3 times	113	28.9
		4 or more	196	50.2
5	Mode of delivery	SVD	355	89.4
		CS	42	10.6
6	Number of PNC during the last birth	Yes	351	88.4
		No	46	11.6
7	The time HIV status is known	Before pregnancy	293	73.8
		During pregnancy	99	24.9
		During delivery	5	1.3
8	Ever started ART?	Yes	397	100
9	Time of ART initiation	Before pregnancy	292	73.6
		During pregnancy	90	22.7
		During delivery	15	3.8
10	HIV status disclosure to spouse	Yes	364	91.7
		No	33	8.3

### Infant health-related factors

**Fig. 1:**
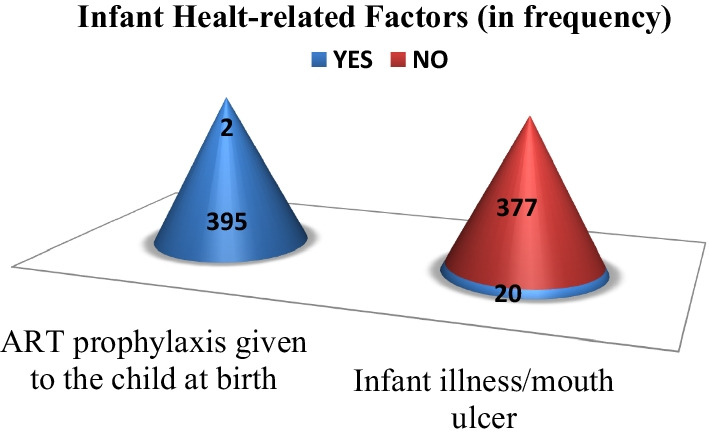
Infant health-related factors among study participants attended at public health facilities in Addis Ababa, Ethiopia, 2022

Infant health-related factors are presented in Fig. 1. Nearly all HIV-exposed infants 395 (99.5%) were given ART prophylaxis at birth while the rest two (0.5%) were given later at postnatal visit. Five percent of HIV-exposed infants had history of infant illness related to mouth ulcer.

### Magnitude of appropriate infant feeding practice (IFP)

**Fig. 2:**
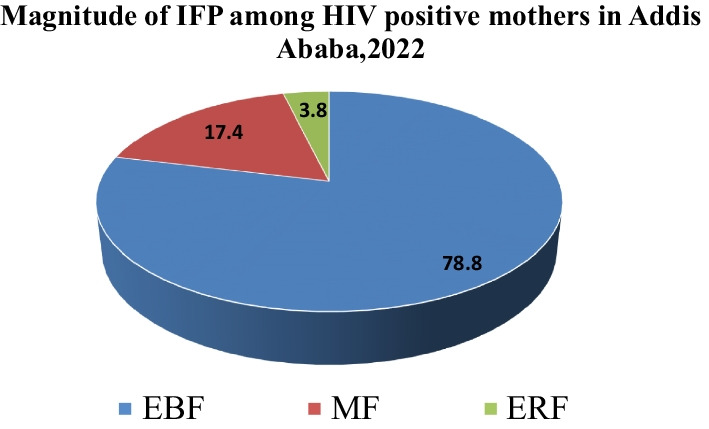
Magnitude if infant feeding practice among HIV-positive mothers attended at public health facilities in Addis Ababa, Ethiopia, 2022. *Note*: EBF (exclusive breast-feeding), ERF (exclusive replacement feeding) and MF mixed feeding

In the current study setting, the magnitude of appropriate infant feeding practice among HIV-positive mothers was 82.6% [95% CI 80.9–88.2]. The remaining 17.4% were practiced inappropriate or not in the recommended way (Fig. 2).

### Knowledge of HIV-positive mothers on PMTCT

Knowledge of HIV-positive mothers on PMTCT was assessed using eleven questions, and 298 (75%) of the study participants were responded more than median in favor of feeding practice (Table 3).

**Table 3:** Knowledge of HIV-positive mothers who had infants with aged 0–6 months in public health facilities in Addis Ababa, Ethiopia, 2022 (*n* = 397)

Questions to answer the variable	Category	Frequency	%
Does HIV-positive mother transmit the virus to her baby during pregnancy, delivery and breast-feeding pregnancy?	Yes	391	98.5
	No	6	1.5
Is it important to initiate breast-feeding within 1 h after birth?	Yes	362	91.2
	No	35	8.8
Feeding only breast milk in the first 6 months helps boost the child immunity	Yes	390	98.2
	No	7	1.8
Can exclusive breast-feeding reduce the risk of diarrhea?	Yes	391	98.5
	No	6	1.5
Growth patterns of exclusively breast-fed infant/s differ from non-exclusively breast-fed?	Yes	383	3.5
	No	14	96.5
How long exclusive breast-feeding should be continued?	Yes	377	95
	No	20	5
Counseled on infant feeding options?	Yes	381	96
	No	16	4
Counseled on infant feeding options during ANC visits?	Yes	315	79.3
	No	82	20.7
Counseled on infant feeding options during delivery visits?	Yes	225	56.7
	No	172	43.3
Counseled on infant feeding options during PNC visits?	Yes	318	80.1
	No	79	19.9
Counseled on infant feeding options during PMTCT visits?	Yes	386	97.2
	No	11	2.8

### Attitude toward infant feeding of HIV-positive mothers

See Table 4.

**Table 4:** Attitude of HIV-positive mothers of children 0–6 months attending in public health facilities in Addis Ababa, Ethiopia, 2022 (*n* = 397)

Variable	Frequency, *n* (%)	
	Yes	No
EBF for 6 months is the best choice for infant	344 (86.6)	53 (13.4)
EBF is not good since it transmits HIV	79 (19.9)	318 (80.1)
EBF for 6 months is nutritionally complete	384 (96.7)	13 (3.3)
Formula feeding is nutritionally complete	187 (47.1)	210 (52.9)
ERF for 6 months is best choice for infants	82 (20.7)	315 (79.3)
Mixed feeding has risk of HIV infection to infant	328 (82.6)	69 (17.4)
Mixed feeding is always necessary to infants	68 (17.1)	329 (82.9)
Wet nurse is good infant feeding option	6 (1.5)	391 (98.5)
Bottle feeding is good infant feeding option	124 (31.2)	273 (68.8)

### Factors associated with infant feeding of HIV-positive mothers

In this study, the result of bi-variable logistic regression analysis showed that there was an association between appropriate infant feeding practice and age of mother (40–44), educational status of mother, knowledge of PMTCT and attitude of HIV-infected mothers toward infant feeding at *p* value of < 0.2. However, after controlling of all possible confounders in multivariable logistic regression analysis, results revealed that households income, knowledge of HIV-positive mothers toward infant feeding and the attitude of mothers toward infant feeding were statistically significant factors associated with infant feeding practice among HIV-positive mothers of children 0–6 months. Those mothers who had HH monthly income ≥ 6001 Ethiopian birr were more likely 1.6 times had an appropriate infant feeding practice than those who have least HH monthly income [AOR = 1.62(1.33,5.14)]. Those mothers who had good knowledge of appropriate infant feeding practice were 1.26 times [AOR = 1.26(1.11, 3.34)] more likely to appropriate infant feeding practice than those with poor knowledge. Similarly, HIV-positive mothers who had a favorable attitude toward infant feeding practice were 1.71 times [AOR = 1.71(1.01, 2.92)] more likely associated with infant feeding practice when compared to those who had an unfavorable attitude (Table 5).

**Table 5:** Binary logistic regression analysis showed the association between appropriate infant feeding practice with other factors among HIV-infected mothers of children 0–6 months attended at Public health facilities in Addis Ababa, Ethiopia, 2022 (*n* = 397)

Predictor variable	Category	Infant feeding practice		Strength of association		*p* value
		Good	Poor	COR (95% CI)	AOR (95% CI)	
Age of mother	20–24	20	13	1	1	
	25–29	76	71	0.69 (0.24,3.5)	1.04 (0.23,4.8)	0.96
	30–34	73	56	0.8 (0.41,4.5)	1.01 (0.26,3.9)	0.98
	35–39	31	44	0.45 (0.23,3.67)	0.89 (0.23,3.5)	0.87
	40–44	7	6	0.75 (0.54,7.6)	1.7 (1.22,6.8)	0.44
Maternal educational status	Illiterate	6 (1.5)	3 7 (9.3)	1	1	
	Literate	151 (38.0)	203(51.1)	0.7 (0.47,1.0)	2.5 (0.91,6.9)	0.073
Household monthly income	< 3000	99	66	1	1	
	3001–6000	61	54	0.75 (0.2,1.6)	0.53 (0.27,1.01)	0.054
	≥ 6001	42	75	0.37 (0.18,0.81)	1.6 (1.33,5.14)	**0.024**
Had support on infant feeding	Yes	128	158	0.38 (0.24.0.61)	0.9 (0.51,1.6)	0.73
	No	75	36	1	1	
Occupation of mother	Governmental	31	55	2.35 (1.37,3.99)	1.92 (1.11,4.12)	0.059
	Private	61	58	0.78 (0.2,1.6)	0.8 (0.47,1.41)	0.45
	Un employed	110	82	1	1	
Disclosure of HIV status	Yes	181	183	1.73 (0.8,3.96)	1.1 (0.45.2.8)	0.79
	No	12	21	1	1	
Knowledge	Good	176	122	3.2 (0.72, 6.24)	1.26 (1.11,3.34)	**0.001**
	Poor	31	68	1	1	
Attitude	Favorable	159	124	2.9 (0.8,5.54)	1.71 (1.01,2.92)	**0.045**
	Unfavorable	35	79	1	1	

## Discussion

In the current study, the magnitude of appropriate infant feeding practice among HIV-positive mothers was (82.6%). In this study, appropriate feeding practice was measured according to the WHO recommendation to prevent mother-to-child transmission of infection [10]. On the bases of this context, appropriate infant breast-feeding practices was measured by including both exclusive breast-feeding (78.8%) and exclusive replacement feeding (3.8%). Accordingly, the magnitude of appropriate infant feeding practice among HIV-positive mothers was consistent with the study finding reported from Gondar (83.8%) [5] and Uganda 79.6% [23]. This might be due to the similarity of the study population, the study settings and the socioeconomic status of the population.

On the other hand, the current research finding showed a relatively slightly higher proportion EBF than the national target set in Ethiopia (70%) by 2020 [20]. In addition, the current study showed higher prevalence of appropriate infant feeding practice than the research finding conducted in India (44%), South Africa (27%) and Ibadan (50.8%) [24, 25], respectively. The possible reason for the difference might be due to the participants’ reliance on replacement feeding, socioeconomic status differences of the study population. The previous study from Addis Ababa reported that the proportion of exclusive breast-feeding practice was (30.6%) [8] was lower than the current study probably to the same reason that mothers were recommended to commercial infant formula for fear of mother-to-child HIV transmission by then. In this study, ERF was 15 (3.8%), which are lower than in the study conducted in eastern Uganda (8.5%) and even Gondar (5.7%) [5, 22] and much lower than what was reported from India (51.3%), South Africa (50%) and Addis Ababa (46.8%) [25–27]. These discrepancies might be due to difference in culture of feeding habit, economic potential, health policy and strategies of intervention. The magnitude of mixed feeding among participants in the present study was 69 (17.4%) which is in line with study done in Kenya (14%), Addis Ababa (15.3%) and Gondar (10.5%) [20, 26, 28]. However, it is comparatively higher than the study conducted in Tigray (6.3%) [16]. This difference might be due to the study population, where in Tigray it has included those who previously seen at the prenatal period and counseled on infant feeding options, this probably helped them to follow the recommended feeding options.

In the current study, HH monthly income above 6001 birr, knowledge and attitude toward infant feeding were significantly associated with infant feeding practice among HIV-positive mothers of children 0–6 months. HIV-positive mothers from a household having an average monthly income of > 6001 were 1.62 times [AOR = 1.6; 95% CI: (1.33, 5.14)] more likely to practice appropriate infant feeding than those mothers from their referent group. Mothers who had good knowledge were 1.26 times [AOR = 1.26(1.11, 3.34)] more likely has appropriate infant feeding practice than those with poor knowledge do. Similarly, HIV-positive mothers who had favorable attitude toward infant feeding practice were 1.71 times [AOR = 1.71(1.01, 2.92)] more likely associated with infant feeding practice when compared to those who had unfavorable attitude. This finding is consistent with studies conducted in Lesotho [28], Botswana [27] and Southern Ghana [29]. This might be because as mothers are empowered through education, the likelihood of their decision to practice recommended feeding increases irrespective of the pressure from partner, family and society as compared to those mothers who are uneducated.

### Strength and limitations

The study was limited to HIV-positive mothers of children below 6 months of age attending public health facilities only and did not incorporate those who were attending private and non-government-owned health facilities. Notwithstanding these drawbacks, we think that the information from our study is crucial for making decisions about exclusive breast-feeding among women with HIV in the current study area and the nationwide as well.

## Conclusions and recommendations

Hence, the current study area is the capital city of the Ethiopia, where relatively educated population lived in, there is an opportunity for better income, and appropriate infant feeding practice among HIV-positive mothers was found slightly higher than even the overall national target (70%) that was planned by 2020. Therefore, different stake holders and the country itself should develop strategic plan to excel females’ education coverage to fully eradicate mother-to-child transmission of diseases.
